# Metasurface loaded multi-port tunable silicon–graphene based THz antenna with LHCP/RHCP characteristics, low-RCS and low-coupling features

**DOI:** 10.1038/s41598-026-56382-z

**Published:** 2026-06-19

**Authors:** Dinesh Kumar Prabhakar, Krishna Kanth Varma Penmatsa, Khushboo Pachori, Ravikiran Huliyurdurga Nagesh, Shivesh Tripathi, Kuldip Singh, Ashish Pandey

**Affiliations:** 1 Department of Electronics and Computer Engineering, National Institute of Advanced Manufacturing Technology, Ranchi, Jharkhand 834003 India; 2 Department of Electronics and Communication Engineering, Sagi Rama Krishnam Raju Engineering College, Bhimavaram, Andhra Pradesh 534204 India; 3 School of Engineering and Technology, Sanjeev Agrawal Global Educational University, Bhopal, Madhya Pradesh 462022 India; 4 Department of Electronics and Communication Engineering, BGS Institute of Technology, Adichunchanagiri University, BG Nagara, Karnataka 571448 India; 5 Department of Electronics and Communication Engineering, G L Bajaj Institute of Technology and Management, Greater Noida, Uttar Pradesh India; 6 Department of Electronics and Communication Engineering, Sant Longowal Institute of Engineering and Technology, Longowal, Punjab India; 7 Department of Data Science and Engineering, Manipal University Jaipur, Jaipur, Rajasthan India

**Keywords:** THz antenna, Graphene antenna, Circular polarization, Low-RCS MIMO antenna, Engineering, Optics and photonics, Physics

## Abstract

The design of a two-terminal antenna with decreased radar cross section (RCS) characteristics and polarization variety is shown in this work. By using stair-aperture feed to excite a cylindrical silicon dielectric resonator, circular polarization is accomplished. An aperture arrangement that is mirror-oriented between terminal-1 and 2 improves isolation by 30 dB. A frequency selective surface based on a split-ring resonator (SRR) is positioned between the twin ports and around the antenna structure in order to reduce monostatic RCS (MRCS). The radiator functions between 2.45 and 3.35 THz, with a 3-dB axial ratio between 2.65 and 3.15 THz. In the operational band, the MRCS is lowered by around 30 dBsm. The suggested design is well suited for radar applications in THz regime due to its broadside radiation and advantageous diversity characteristics.

## Introduction

Future applications leveraging the extensive bandwidth of the terahertz (THz) spectrum will require significantly faster data transmission, making THz antennas an increasingly prominent focus in current antenna research^1,2^. Recent technological advancements have enabled the development of various THz antenna designs, including ceramic-based, metallic, and tunable graphene-based configurations^3–5^. In certain military applications, circular polarization (CP) and decreased radar cross section (RCS) are required together. RCS reduction helps to minimize detectability^6^, whereas CP guarantees orientation-independent performance^7^.

Improvements in high-speed internet connection are being driven by the need for greater data transmission rates, and MIMO systems are anticipated to be crucial in fulfilling these demands^8^. Multiple antenna integration is necessary for these systems, and THz applications are having a growing impact on MIMO antenna development. A four-port S-shaped slot-coupled composite radiator with circular polarization and multidirectional far-field patterns in the 2.51–2.75 THz range was suggested by Kumar et al.^9^. It operates between 2.49 and 2.85 THz. A two-port orthogonally oriented S-shaped printed-line-driven silicon ceramic antenna with circular polarization support throughout its band was created by Harish et al. and operates between 2.49 and 3.29 THz^10^. Using a frequency selective surface (FSS), Yadav et al. created a two-port stair-shaped silicon dielectric antenna that covers 2.35–3.15 THz. The radiation patterns from the various ports are slanted to ± 45°^11^. A circular-aperture-fed silicon dielectric antenna with two ports was presented by Voruganti. It operates between 2.41 and 2.8 THz and uses a MS to transform linear polarization into circular polarization^12^. In order to achieve circular polarization within its operational spectrum, Verma et al. introduced a two-port silicon dielectric antenna supplied by a swastik aperture that covers the frequency range of 2.4–3.2 THz^13^. Recent research on graphene-based terahertz (THz) antennas has focused on improving tunability, isolation, and multifunctional MIMO performance for future 6G and nano communication applications^14–19^. Studies have demonstrated self-diplexing/self-multiplexing characteristics, frequency agility, and high isolation enhancement using graphene biasing, slotted structures, and defected ground techniques^14,15^. Overall, graphene-enabled THz antennas offer compact, reconfigurable, and high-performance solutions with improved bandwidth, filtering response, and mutual coupling reduction for advanced wireless systems^16–19^. Recent studies on terahertz (THz) antennas have also emphasized broadband operation, circular polarization, beam steering, and high-gain array configurations. Researchers have utilized graphene materials, polyimide substrates, hybrid couplers, and array techniques to enhance impedance bandwidth, radiation efficiency, gain, and polarization characteristics in THz antennas^20–25^.

Despite significant progress in THz antenna research, several important challenges still exist in currently reported THz MIMO and graphene-based antenna designs. The major limitations observed in existing literature are summarized as follows:

- Most reported THz antennas focus only on either frequency tunability or circular polarization, while simultaneous implementation of multiple functionalities remains limited.
- Achieving high isolation between closely spaced THz MIMO antenna elements is still a major challenge.
- Many existing THz antennas suffer from narrow axial-ratio bandwidth and limited impedance bandwidth.
- Low radar cross section (RCS) characteristics are rarely considered together with MIMO and circular polarization performance.
- Existing isolation enhancement techniques often increase structural complexity or degrade radiation performance.
- Limited studies are available on metasurface-assisted RCS reduction in graphene-enabled THz dielectric resonator antennas.
- Practical integration of polarization diversity, reconfigurability, and low-RCS characteristics within a compact THz platform remains insufficiently explored.

To address these challenges, the proposed work introduces a metasurface-loaded silicon–graphene cylindrical dielectric resonator antenna operating in the THz regime. The proposed design simultaneously achieves:

- Wide impedance bandwidth (2.45–3.35 THz),
- Circular polarization with wide 3-dB axial-ratio bandwidth,
- Frequency tunability using graphene chemical potential control,
- Isolation improvement beyond 30 dB using polarization diversity,
- Significant monostatic RCS reduction using SRR-based metasurface loading,
- Excellent diversity performance with low ECC and high DG.

The novelty of the proposed work lies in the simultaneous realization of multiple important characteristics within a compact THz MIMO platform, including graphene-based frequency reconfigurability, circular polarization generation, high isolation through polarization diversity, and low radar cross section (RCS) using SRR-based metasurface loading. Therefore, the proposed antenna provides an integrated solution for next-generation THz wireless communication and stealth applications.

## Antenna arrangement

**Fig. 1:**
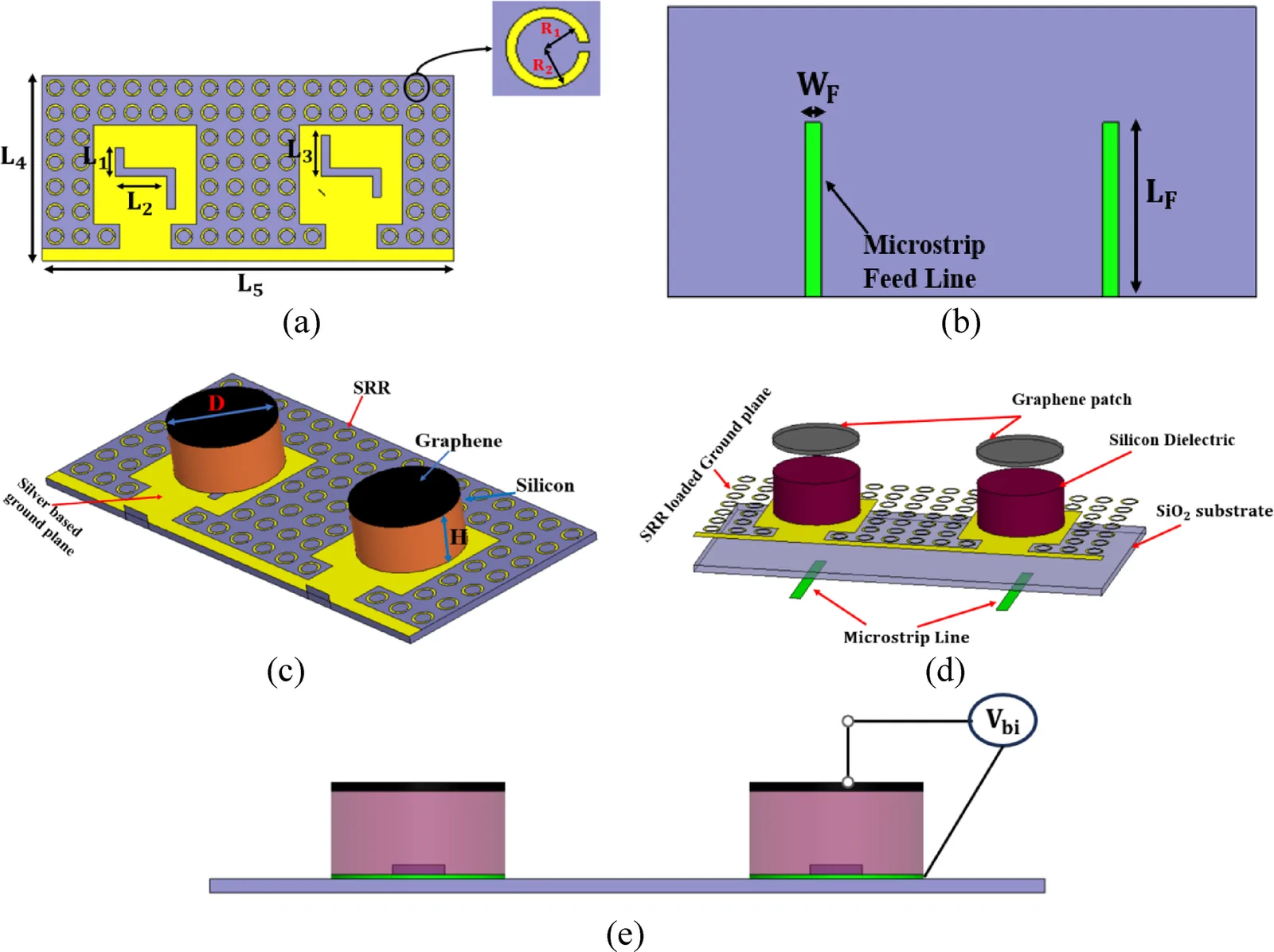
Layout of antenna design (**a**) top view w/o dielectric rod (**b**) microstrip feed line (**c**) isometric view (**d**) layered structure (**e**) biasing mechanism; L_1_ = 4.0; L_2_ = 3.0; L_3_ = 6.0; L_4_ = 22.5; L_5_ = 48.0; L_F_ = 22.0; W_F_ = 2.1; R_1_ = 0.5; R_2_ = 0.75; D = 8.0; H = 8.0 (in µm).

The suggested multi-terminal antenna’s design is shown in Fig. 1. The stair-aperture-coupled silicon ceramic used in the two-terminal design is made on a SiO_2_ substrate (ε_r_ = 3.9) that is 1.6 µm thick. To provide adjustable antenna properties, a 0.34 nm thick coating of graphene may be applied on the silicon dielectric. Silver is used to make the conductive components, which include the ground plane and microstrip feed lines. SRR built MS is made on the upper surface of SiO_2_ substrate. Silicon dielectric material has been selected in the proposed THz antenna design due to its favourable electromagnetic, fabrication, and integration characteristics in the terahertz frequency regime. Silicon possesses relatively high dielectric permittivity ($\varepsilon_{r} \approx 11.2$), which enables strong electromagnetic field confinement and compact antenna dimensions suitable for THz miniaturized structures. Figure 1d shows the layered antenna design as well as Fig. 1e shows the biasing structure for proposed antenna.

In comparison with conventional substrate materials such as RT/Duroid, Taconic, Arlon, quartz, glass, and polyimide, silicon provides improved compactness and enhanced compatibility with semiconductor fabrication technologies including MEMS and CMOS processes. Furthermore, silicon dielectric resonators exhibit efficient radiation characteristics and stable resonant behaviour in the THz regime.

Although substrates such as quartz and glass provide lower dielectric loss, they generally require comparatively larger antenna dimensions due to lower dielectric permittivity. Flexible substrates such as polyimide offer mechanical flexibility but exhibit limited field confinement and comparatively lower integration suitability for compact THz resonator structures.

Graphene has been selected in the proposed THz antenna design due to its unique plasmonic and tunable electromagnetic properties in the terahertz frequency regime. In contrast to conventional metallic conductors such as copper, gold, and silver, graphene behaves as a two-dimensional conductive material whose surface conductivity can be dynamically controlled through chemical potential variation. In the THz regime, graphene supports surface plasmon polariton (SPP) waves that enable strong electromagnetic field confinement and compact antenna dimensions. This plasmonic behavior allows miniaturization of the antenna structure while simultaneously enabling frequency reconfigurability through electrostatic biasing.

The Drude formulation, which is written as^14^, is used to represent the antenna’s dispersive behaviour.

1 $${\upvarepsilon}_{{{\mathrm{Ag}}}} = {\upvarepsilon}_{0} \left[ {{\upvarepsilon}_{{\mathrm{r}}} - \frac{{{\mathrm{f}}_{1}^{2} }}{{\left\{ {{\mathrm{f}} + {\mathrm{if}}_{2} } \right\}}}} \right]$$

The plasma frequency and collision frequency are denoted by f_1_ and f_2_, respectively, in the equation above. The theoretical values for f_1_ and f_2_ for silver utilized in THz antenna applications are 4.25 THz and 2265 THz, respectively^26^. Due to the extremely small physical dimensions of THz antennas, conventional coaxial feeding techniques used at microwave frequencies are not suitable for practical implementation. In the proposed design, the antenna is excited using an aperture-coupled planar microstrip feeding structure fabricated through semiconductor-compatible lithographic processes. For experimental characterization, the proposed antenna may be excited using THz on-wafer probe stations, photomixing-based THz sources, or integrated THz transceiver circuits connected to the planar feed structure.

### Design guidelines of proposed antenna

The proposed THz antenna design was developed through a systematic optimization process to simultaneously achieve wide impedance bandwidth, circular polarization, high isolation, and low-RCS characteristics.

- Initially, a single cylindrical silicon dielectric resonator excited through a rectangular aperture was investigated. However, this configuration provided limited bandwidth and failed to generate circular polarization.
- To improve the impedance and axial-ratio bandwidth, the rectangular aperture was modified into a stair-shaped slot with unequal vertical strip lengths. The stair-slot structure introduces two orthogonal degenerate modes with approximately 90° phase difference, which is essential for circular polarization generation.
- In the next stage, a graphene coating was incorporated over the cylindrical dielectric resonator to realize frequency tunability through chemical potential variation. The graphene layer enables dynamic control of the resonant frequency without significantly affecting radiation performance.
- To realize a MIMO configuration with enhanced isolation, two antenna elements were arranged in mirror-image orientation. This arrangement improves polarization diversity and significantly suppresses mutual coupling between the ports.
- Finally, an SRR-based metasurface was integrated around and between the antenna elements to reduce monostatic RCS and further suppress surface-wave-induced coupling. The metasurface introduces impedance discontinuities and improves stealth characteristics without degrading antenna bandwidth or circular polarization performance.

## Comprehensive antenna investigation

**Fig. 2:**
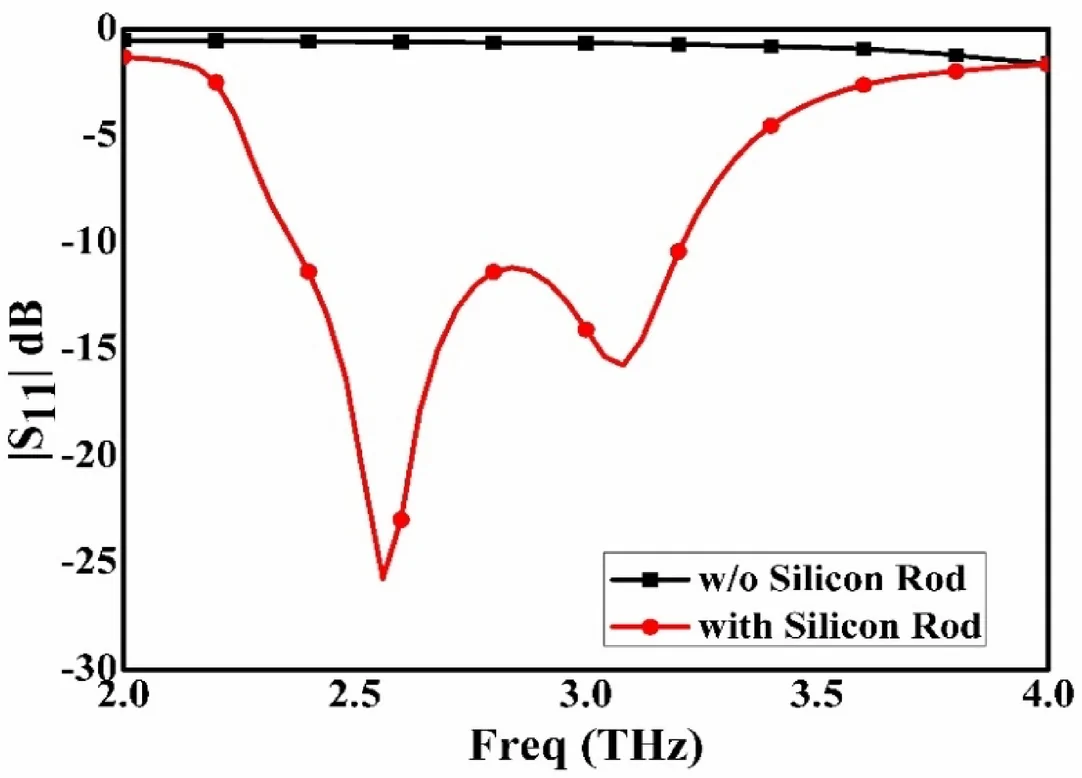
Comparison of |S11| characteristics in the presence and absence of cylindrical silicon dielectric resonator.

Using HFSS software, this part offers a detailed examination of the suggested antenna design. The |S_11_| characteristics with and without the cylindrical silicon dielectric resonator are shown in Fig. 2. Dual resonances at 2.5 THz and 3.1 THz are found, when the silicon dielectric fed by a stair aperture is in place. The |S_11_| stays near zero when the Si dielectric is not there. The improvement in return loss observed after introducing the cylindrical silicon dielectric resonator is mainly attributed to enhanced electromagnetic field confinement and improved impedance matching between the feed structure and radiating element.

**Fig. 3:**
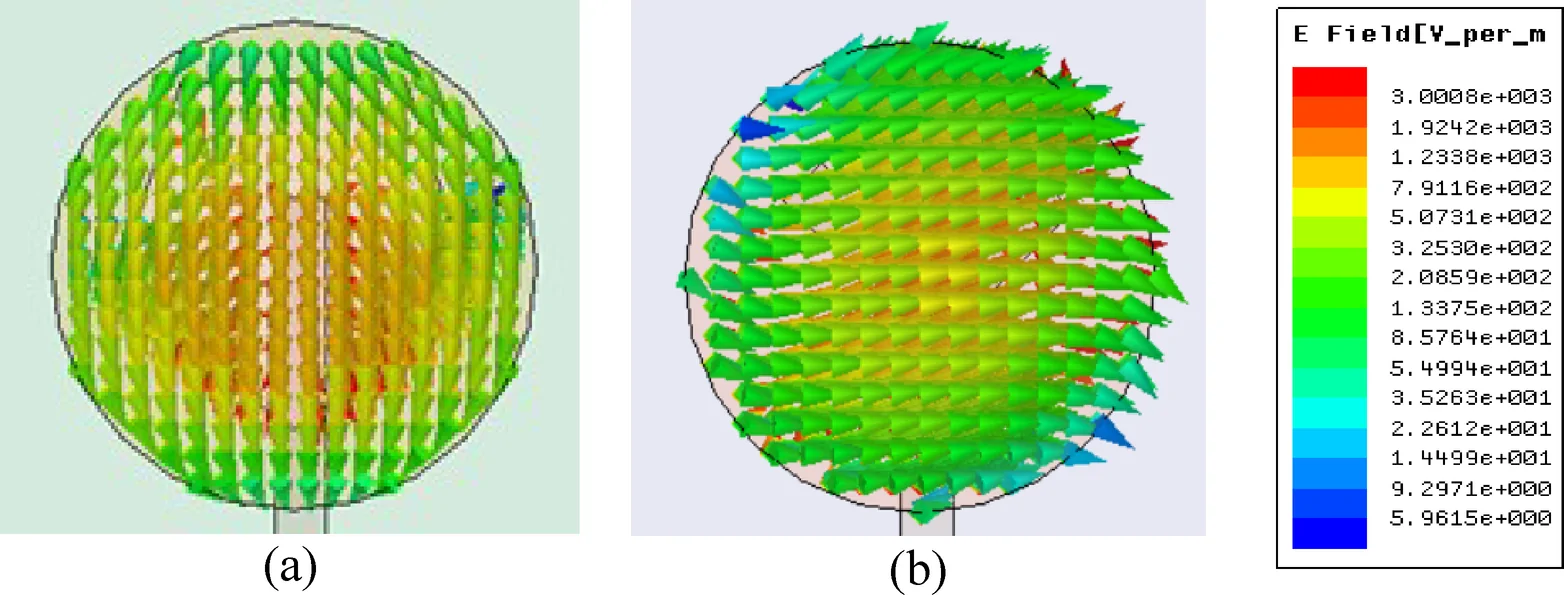
E-field distribution on cylindrical silicon dielectric resonator (**a**) 2.5 GHz and (**b**) 3.1 GHz.

The electric-field distribution inside the cylindrical silicon dielectric resonator is analysed to study the excited resonant modes at 2.5 THz and 3.1 THz, as seen in Fig. 3. The stimulation of the ${\mathrm{HEM}}_{{11{\updelta }}}^{{\mathrm{X}}}$ and ${\mathrm{HEM}}_{{11{\updelta }}}^{{\mathrm{Y}}}$ mode inside the silicon dielectric is confirmed by the observation of the field lines^27^. The stair aperture, which acts as a magnetic dipole, is responsible for this mode creation. The following formula^28^ may also be used to mathematically confirm the excitation mechanism:

2 $${\mathrm{f}}_{{\mathrm{r}}} = \frac{{6.324{\mathrm{c}}}}{{2{\pi D}\sqrt {{\upvarepsilon } + 2} }}\left\{ {0.27 + 0.36\left( {\frac{{\mathrm{D}}}{{4{\mathrm{H}}}}} \right) + 0.02\left( {\frac{{\mathrm{D}}}{{4{\mathrm{H}}}}} \right)^{2} } \right\}$$

In the equation above, ‘ε’ stands for the silicon dielectric’s dielectric constant, while ‘D’ and ‘H’ stand for the dielectric’s diameter and height, respectively. Equation (2) yielded an estimated resonance frequency of 2.81 THz, which is in good accord with the outcome of the simulation.

**Fig. 4:**
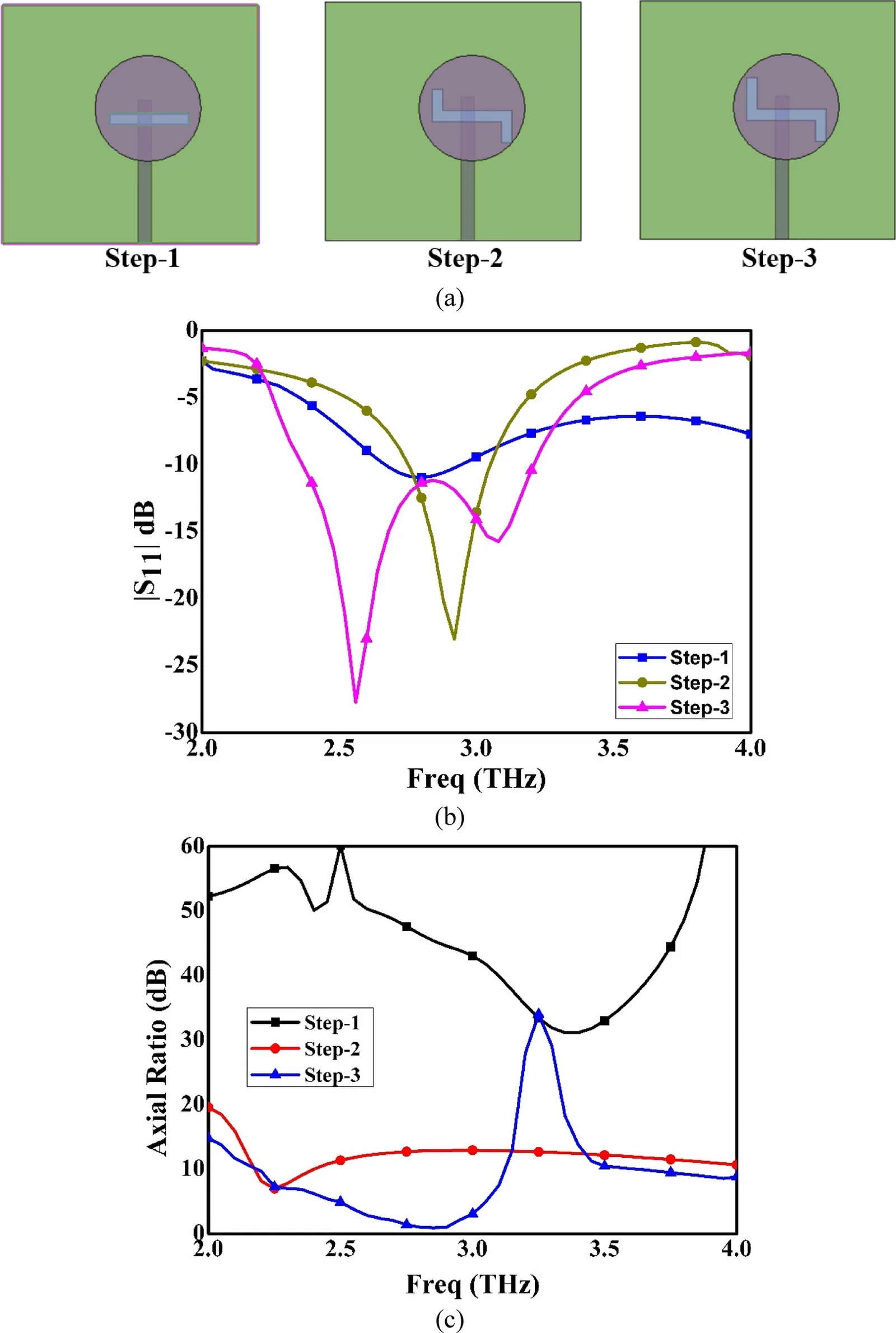
(**a**) Evolution of single port antenna design. (**b**) Graph of |S_11_| for various steps involved in single port antenna design. (**c**) Graph of AR for various steps involved in single port antenna design.

Figure 4a shows the different antenna steps involved to design single port structure. Step-1 contains horizontal slot coupled dielectric. Step-2 and step-3 contain the stair shaped slot with equal and unequal vertical strips fed dielectric. The |S_11_| fluctuation for these steps is shown in Fig. 4b. It can be seen from Fig. 4b that the impedance bandwidth has been improved in case of stair slot (unequal vertical strips) fed cylindrical silicon dielectric resonator. This is due to orthogonal mode development inside silicon ceramic. Figure 4c displays the AR fluctuation for all three above cases. It is clear that in the 2.51–3.11 THz range, the stair (unequal vertical strips) slot attains an axial ratio below 3 dB. Stair shaped aperture creates orthogonal modes inside the dielectric rod, while change in arm length produces the path delay between the orthogonal field elements. It produces the 90^0^ phase shift between field components. This arrangement satisfies the essential condition for the formation of circularly polarized waves^7^.

Reconfigurability is made possible by depositing a graphene layer on top of the silicon ceramic. The following equation^10^ may be used to represent the intra-band conductivity of graphene, which can be changed by adjusting the chemical potential (µc):

3 $$\sigma_{intra} \left( {\omega ,\mu_{c} ,{\Gamma },T} \right) = - j\frac{{e^{2} K_{B} T}}{{\pi \hbar^{2} \left( {\omega - j2{\Gamma }} \right)}}\left( {\frac{{\mu_{c} }}{{K_{B} T}} + 2ln\left( {e^{{ - \frac{{\mu_{c} }}{{K_{B} T}}}} + 1} \right)} \right)$$

The Boltzmann constant, operating temperature, chemical potential, and scattering rate are denoted by the variables K_B_, T, µ_c_, and Γ, respectively, in this equation. In the present work, graphene conductivity was modelled using the Kubo conductivity formulation considering intra-band conductivity contribution dominant in the THz regime. The chemical potential ($\mu_{c}$) was varied from 0.1 to 0.7 eV to investigate the frequency reconfigurability characteristics of the proposed antenna. The relaxation time (*τ*) of graphene was selected as 0.1 ps. The operating temperature was assumed to be 300 K.

In HFSS, graphene was implemented using an impedance boundary condition assigned to an ultra-thin conductive layer. The equivalent complex surface impedance corresponding to the graphene conductivity was manually incorporated into the simulation environment. Similarly, in CST Microwave Studio, graphene was modelled using a surface impedance sheet or thin conductive layer with user-defined frequency-dependent conductivity parameters. The conductivity values corresponding to different chemical potentials were defined through material equations and assigned to the graphene surface. In the proposed structure, the tunability of graphene is achieved through electrostatic biasing, where the chemical potential (Fermi level) is controlled by an external gate voltage. Practically, instead of applying bias through the entire substrate thickness, a localized gating structure can be employed using a thin dielectric spacer such as Al_2_O_3_ or HfO_2_ beneath the graphene layer.

**Fig. 5:**
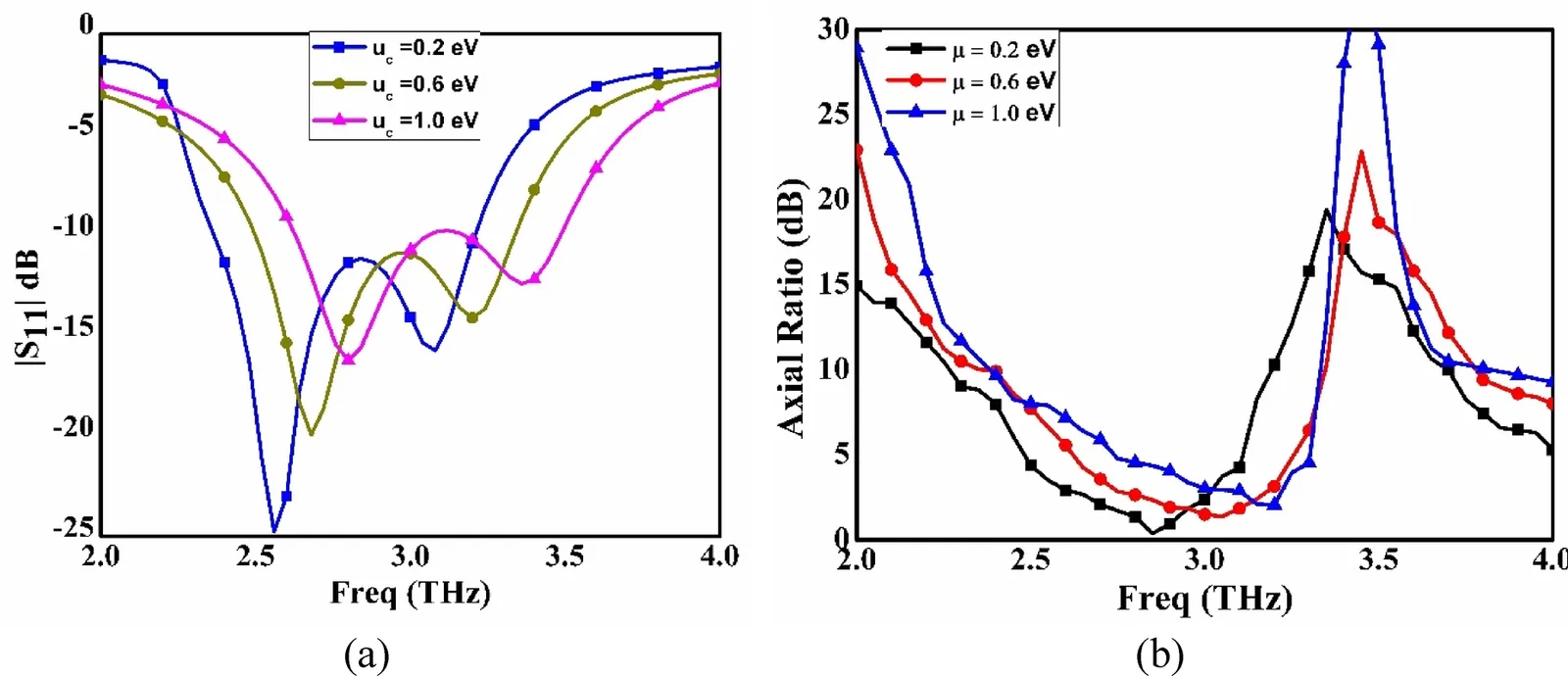
Effect of graphene layer chemical potential (**a**) |S_11_| (**b**) AR.

By varying its electrostatic bias, the graphene layer’s chemical potential may be adjusted. The reflection coefficient (∣S_11_∣) response of the single-port array is shown in Fig. 5a. It is evident that ∣S_11_∣ resonance changes toward higher frequencies, when μ_*c*_ increases. As the chemical potential increases, the carrier concentration and conductivity of graphene also increase, resulting in a shift of resonant frequency toward higher frequencies. The equivalent AR fluctuation is seen in Fig. 5b, whereas μ_*c*_ rises, the 3-dB axial ratio band also shifts to higher frequencies. These findings verify that frequency reconfigurability is supported by the suggested THz radiator. Table 1 lists the performance variation of proposed radiator with change in chemical potential. The results indicate that increasing graphene chemical potential shifts the operating band toward higher frequencies while improving impedance and axial-ratio bandwidth performance. The resonant frequency shifts toward higher frequencies because increasing graphene conductivity modifies the effective propagation constant and reactive impedance of the antenna structure.

**Table 1:** Performance variation with graphene chemical potential.

Chemical potential ($\mu_{c}$)	Approx. bias voltage	Operating range (THz)	Resonant frequency (THz)	Impedance BW (%)	3-dB AR BW (%)
0.1 eV	Low	2.30–2.75	2.45	24.6	7.2
0.3 eV	Moderate	2.45–2.90	2.68	27.8	8.5
0.5 eV	Moderate-High	2.75–3.20	2.92	30.4	9.7
0.7 eV	High	2.90–3.35	3.15	32.5	10.8

**Fig. 6:**
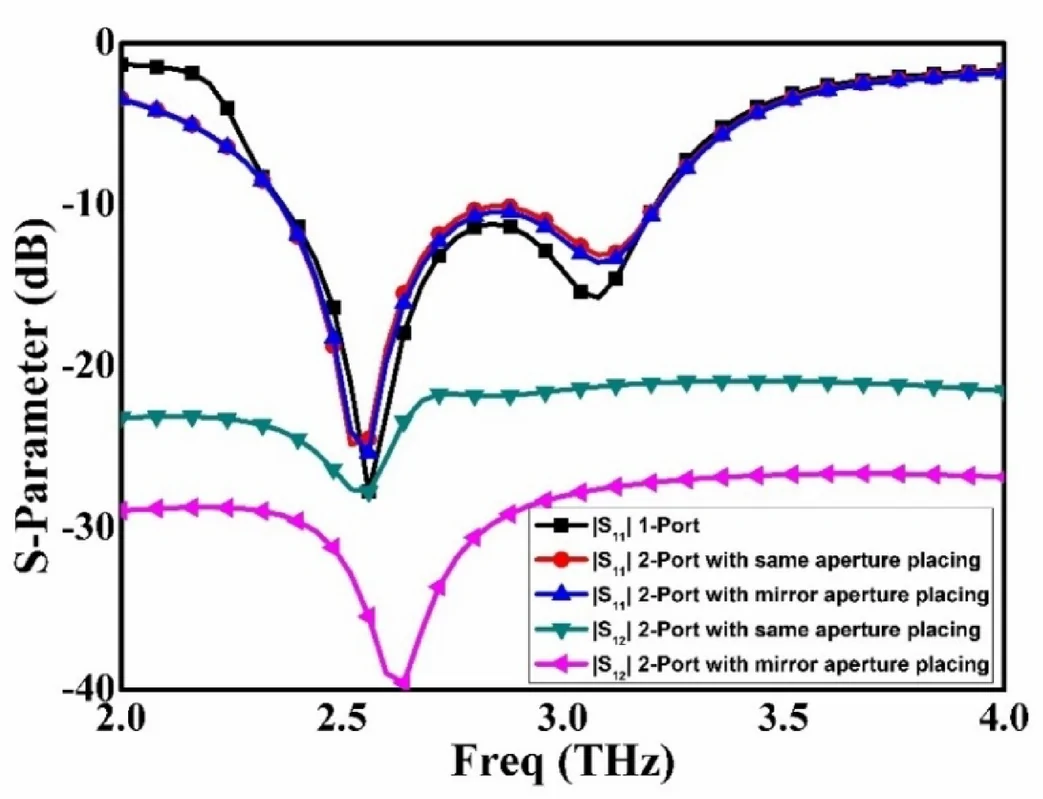
S-parameter response for 1-port and 2-port configurations.

The S-parameter properties for 1-port and 2-port systems are shown in Fig. 6. Two situations are considered for the 2-port case: (a) both ports have the same slot position, and (b) the slot orientations are mirror images. As can be seen, |S_11_| is almost the same in all three layouts. The mirror-image configuration, on the other hand, greatly reduces mutual coupling, reaching levels below − 30 dB. The polarization diversity effect is responsible for this improvement^29^.

**Fig. 7:**
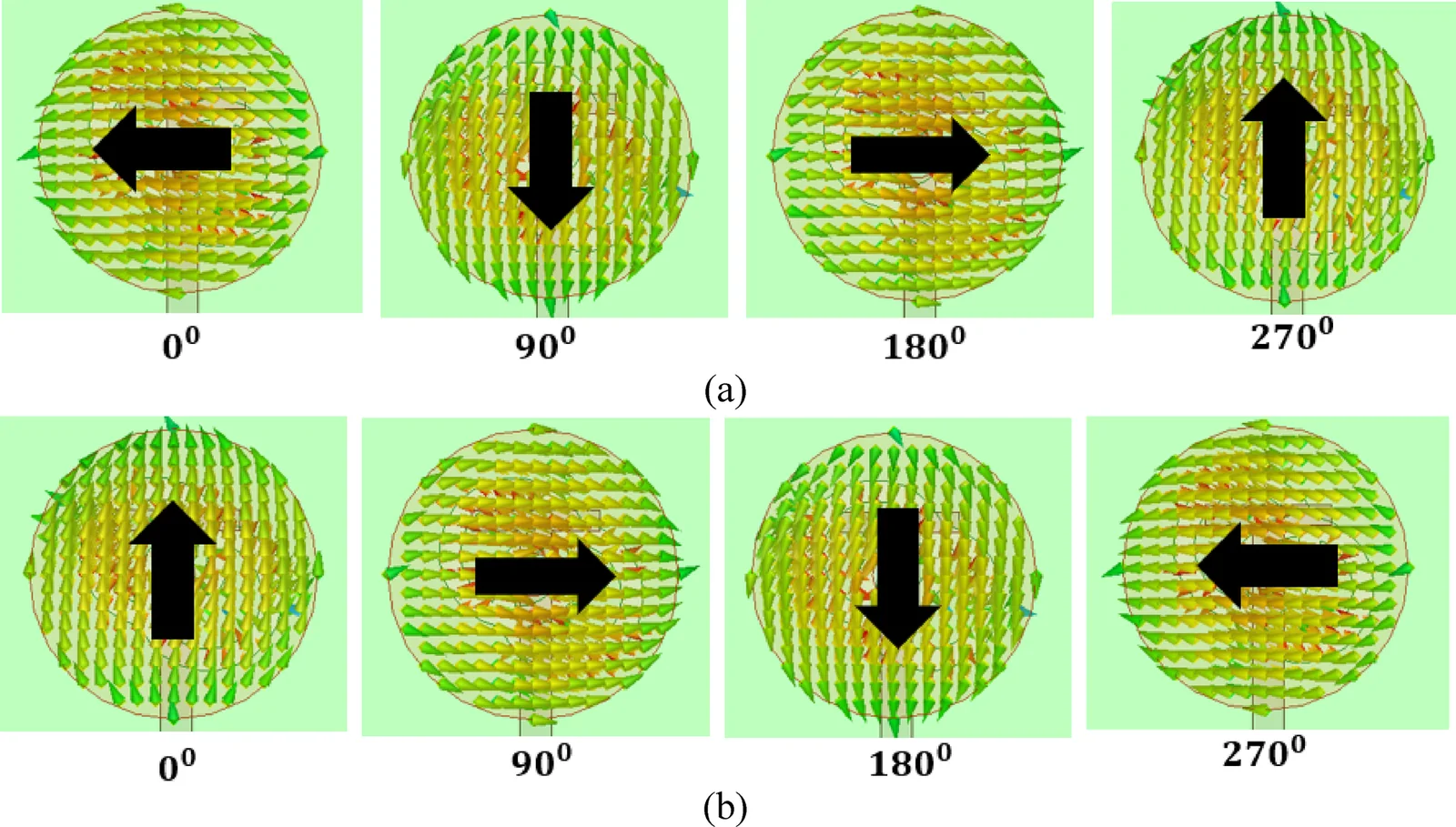
E-field rotation with different phase at 2.8 THz (**a**) Port-1 (**b**) Port-2.

The electric field’s rotation at 2.8 THz for various phase angles with port-1 and port-2 is shown in Fig. 7a and b. The creation of circularly polarized waves is confirmed by these figures, which clearly show that the electric field vectors rotate in a circle. The existence of left-hand circular polarization (LHCP) is indicated by the field’s anticlockwise rotation with port-1. On the other hand, the excitation of right-hand circular polarization (RHCP) waves is confirmed by the clockwise rotation of the electric field with port-2.

**Fig. 8:**
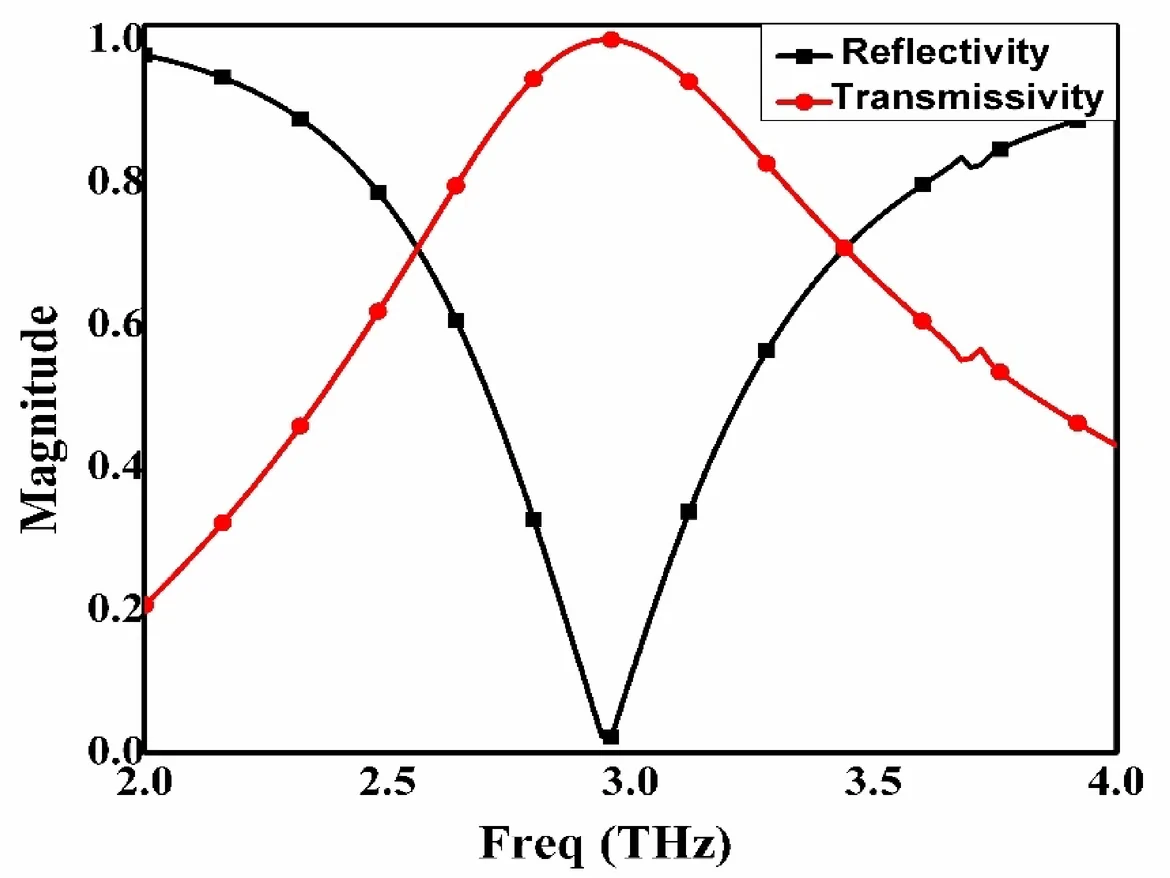
Graph of reflectivity and transmissivity of designed SRR.

The suggested split-ring resonator (SRR) unit cell’s transmission and reflection responses are shown in Fig. 8. The planned unit cell displays band-pass filtering properties within the intended operating frequency range, as shown in the figure. Throughout the operating range, the transmission coefficient stays over 0.8 and the reflection coefficient stays below 0.5, suggesting effective signal transmission with lower reflection losses. The suggested SRR unit cells are positioned regularly on the ground plane of the antenna construction to further improve the antenna performance without affecting the radiator characteristics. While preserving steady radiation performance in the targeted frequency range, this periodic metasurface arrangement enhances the antenna’s electromagnetic behaviour.

**Fig. 9:**
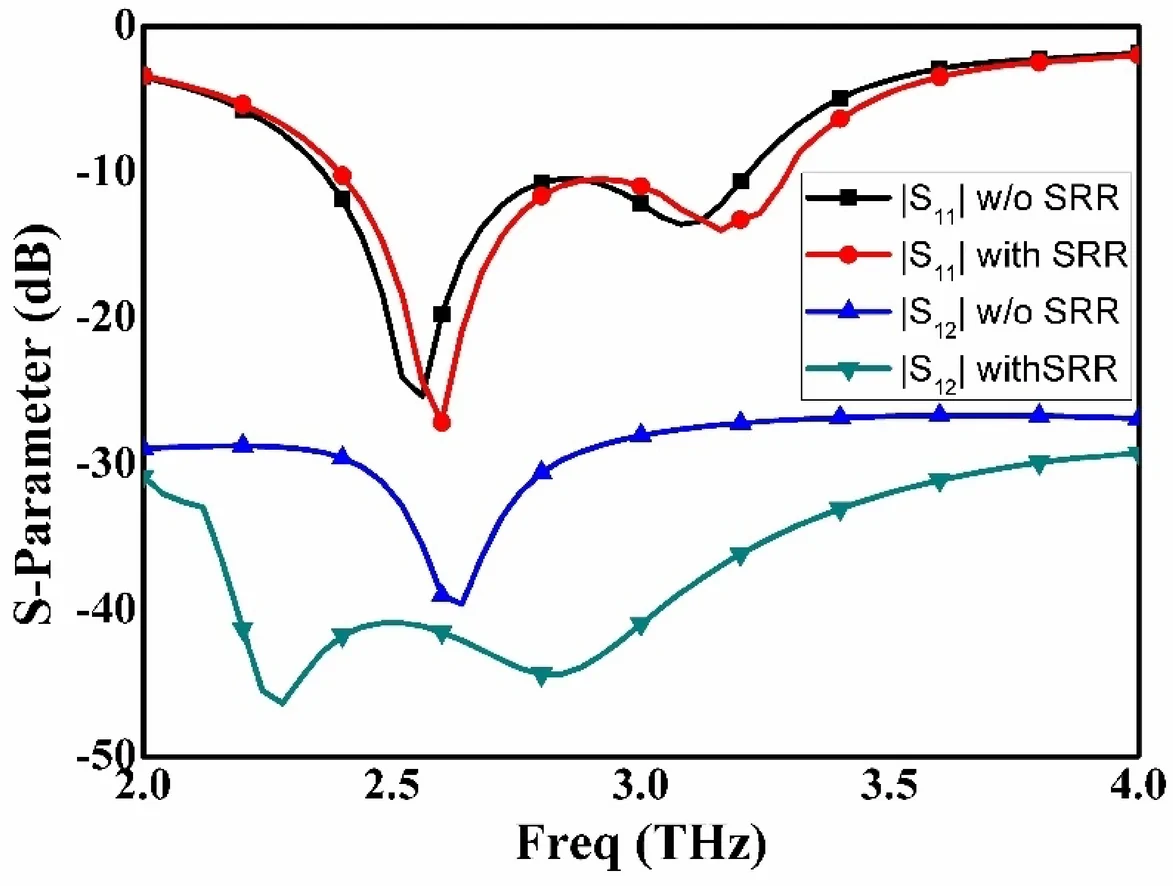
S-parameter response with and without SRR.

The S-parameter response of the planned antenna is illustrated in Fig. 9. It can be observed that the inclusion of the split-ring resonator (SRR) does not significantly affect the |S_11_|. However, the presence of the SRR reduces the coupling within the operating band to below − 35 dB. This reduction is attributed to the discontinuity in surface currents introduced by the SRR structure. The SRR metasurface behaves as an electromagnetic LC resonator where the metallic ring contributes inductive behaviour and the split gap introduces capacitance. Around resonance, strong localized circulating currents are generated within the SRR structure, producing reactive electromagnetic fields that suppress surface-wave propagation and reduce induced mutual currents between adjacent antenna elements. The transmission and reflection responses shown in Fig. 8 indicate that the SRR structure operates as a frequency selective surface (FSS) with engineered impedance characteristics in the operational frequency band. The resonant SRR response introduces impedance discontinuities and stop-band characteristics for coupling modes, thereby significantly improving port isolation.

The centre-to-centre spacing between the two dielectric resonator elements is approximately 0.48λ_0_ at the center frequency of 2.9 THz. Although the antenna elements are placed within relatively compact spacing, isolation greater than 30 dB is achieved through the combined effects of polarization diversity, mirror-image excitation, orthogonal hybrid-mode generation, and SRR-based metasurface decoupling. The mirror-image arrangement produces orthogonal current distributions and opposite circular polarization characteristics between the two ports, thereby suppressing direct electromagnetic coupling. Furthermore, the SRR-based metasurface suppresses surface-wave propagation and introduces impedance discontinuities in the mutual coupling path, significantly reducing induced surface currents between adjacent antenna elements.

**Fig. 10:**
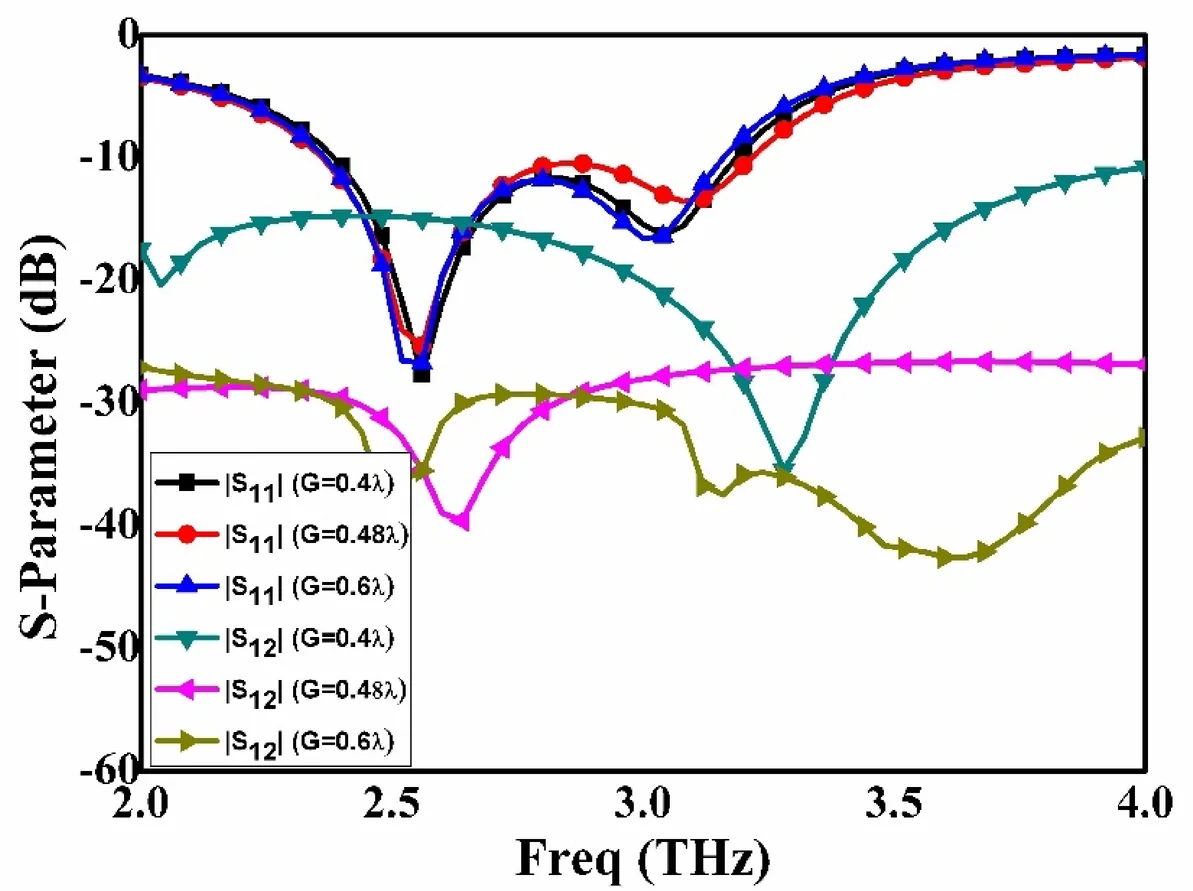
Effect of inter-element spacing on S-parameter characteristics of the proposed THz MIMO antenna.

A parametric analysis of inter-element spacing is also carried out to investigate its influence on impedance matching and mutual coupling characteristics, which is shown in Fig. 10. The results indicate that spacing below 0.4λ_0_ increases near-field coupling, whereas larger spacing slightly improves isolation at the expense of antenna compactness. The proposed spacing of 0.48λ_0_ provides an optimized balance between compact size, impedance bandwidth, and high isolation performance.

**Fig. 11:**
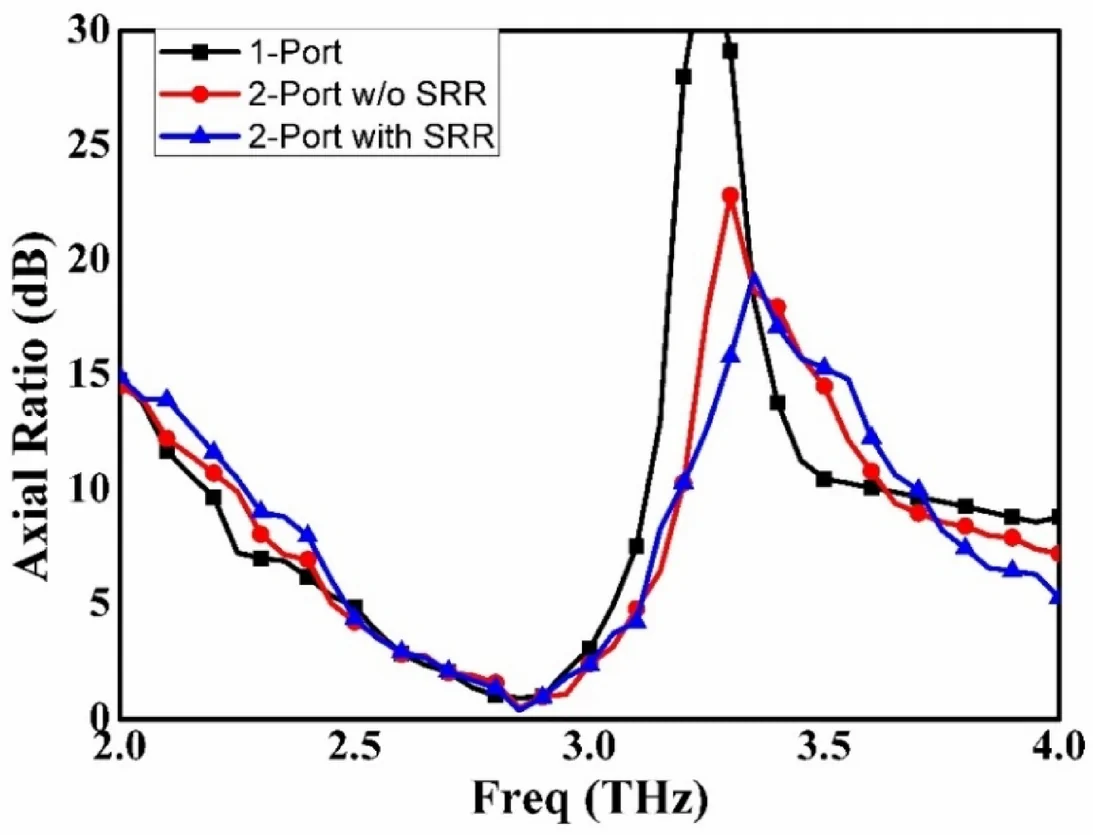
S-parameter response for 1-port and 2-port antenna (with and without SRR).

Figure 11 presents the axial ratio variation for both 1-port and 2-port configurations, with and without SRR. The results indicate that the axial ratio performance remains nearly identical in all three cases, which is advantageous for MIMO antenna applications.

**Fig. 12:**
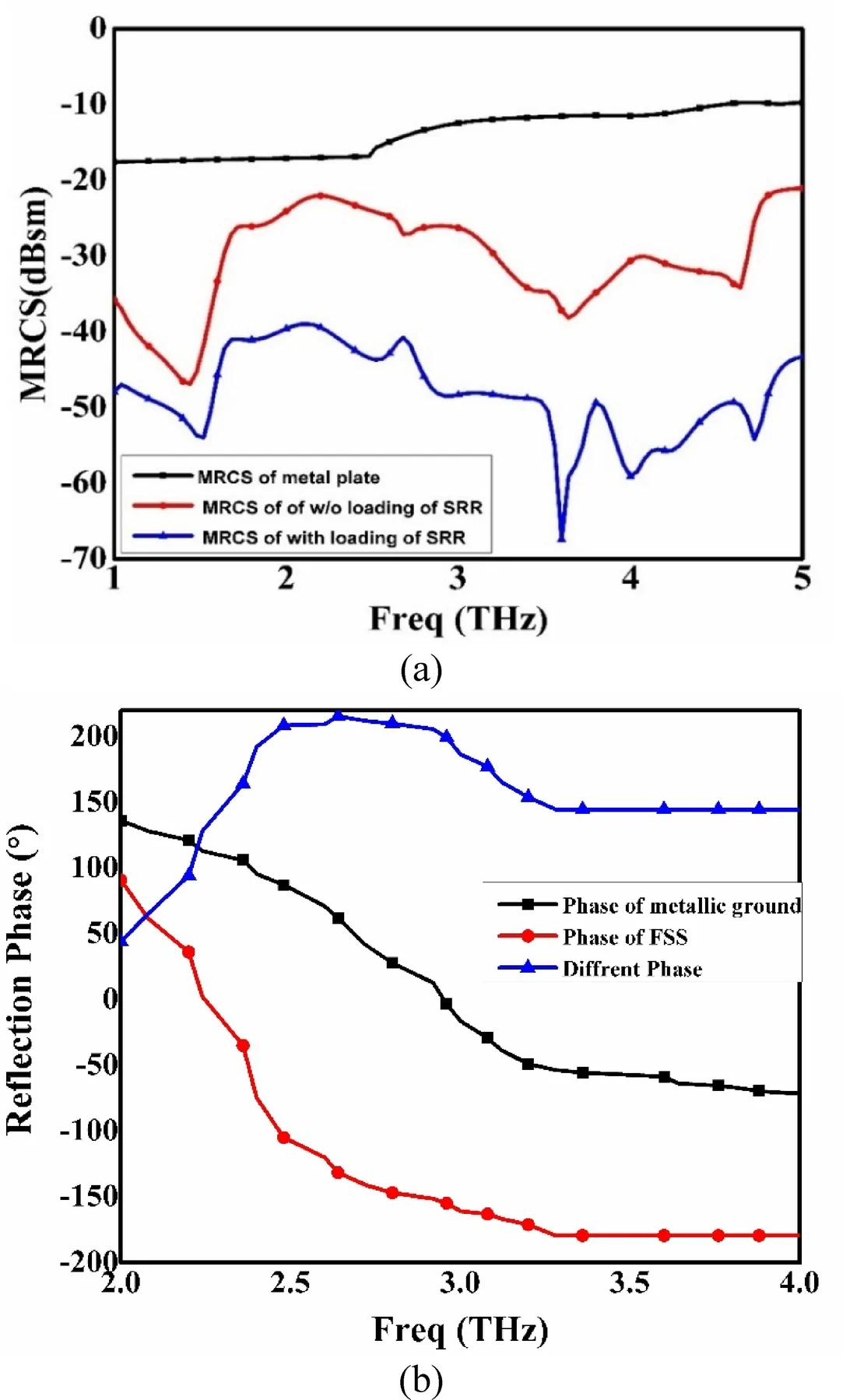
(**a**) Monostatic RCS variation for diverse structural configurations. (**b**) Comparison of reflection phase of FSS and metallic ground.

The monostatic RCS fluctuation of the planned antenna is shown in Fig. 12a with three cases: (i) Antenna installed on a metallic plate; (ii) without SRR loading; (iii) with SRR loading. When SRR is used, the in-band RCS is significantly reduced, falling below –30.0 dBsm, as shown in Fig. 12a. The reduction in RCS can be explained through the reflection phase characteristics of FSS and metallic ground, along with their corresponding phase difference, as illustrated in Fig. 12b. It is evident from the figure that the phase difference between FSS and metallic ground remains within the range of 180° ± 37°. Since the phase difference is approximately 180°, destructive interference occurs between the reflected waves, leading to significant RCS reduction. However, the phase difference is not exactly 180° over the entire operating band, indicating that perfect destructive interference is not achieved in practice. Consequently, the effectiveness of RCS suppression is slightly reduced under practical conditions.

**Fig. 13:**
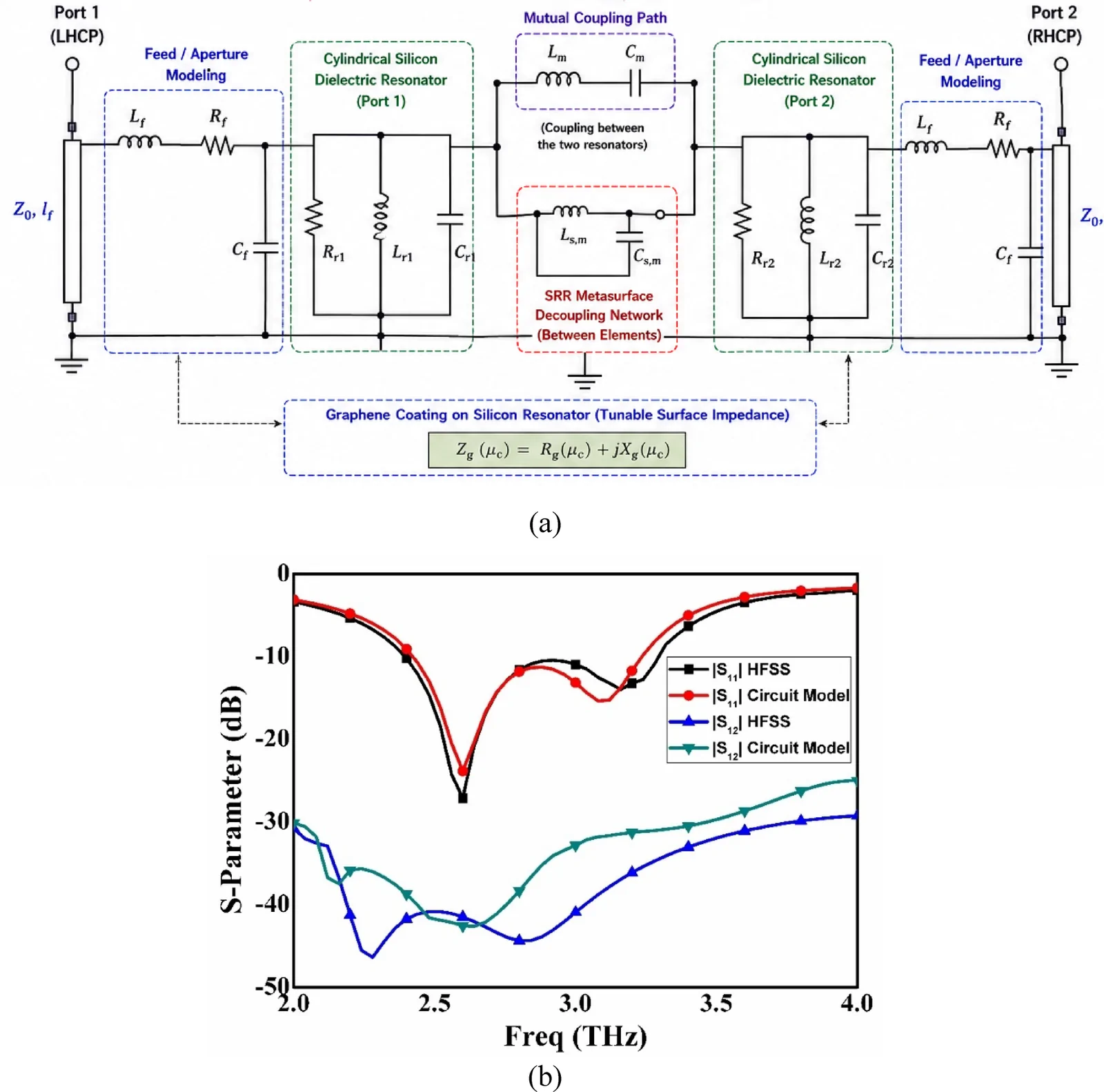
(**a**) Equivalent circuit of proposed THz antenna design. (**b**) Plot of S-parameter got from HFSS and circuit model.

Figure 13a shows the equivalent circuit model of proposed antenna. In the equivalent circuit representation of the proposed antenna, the cylindrical dielectric resonator behaves as a parallel RLC resonator, while the SRR metasurface introduces additional parallel LC branches coupled to the radiating structure. The graphene layer is modelled using tunable complex surface impedance dependent on chemical potential. The SRR-based metasurface introduces reactive impedance loading and suppresses surface-wave-induced mutual coupling between adjacent antenna elements. Near the SRR resonance frequency, the induced counter-reactive currents reduce electromagnetic field leakage and improve isolation characteristics. Furthermore, the reactive compensation introduced by the SRR structure improves impedance matching and contributes toward monostatic RCS reduction. Figure 13b shows the comparison of S-parameter obtained from HFSS and circuit model. It is clearly observed from Fig. 13b that there is good matching between HFSS and circuit model-based outcomes.

## Verification of optimal outcomes

**Fig. 14:**
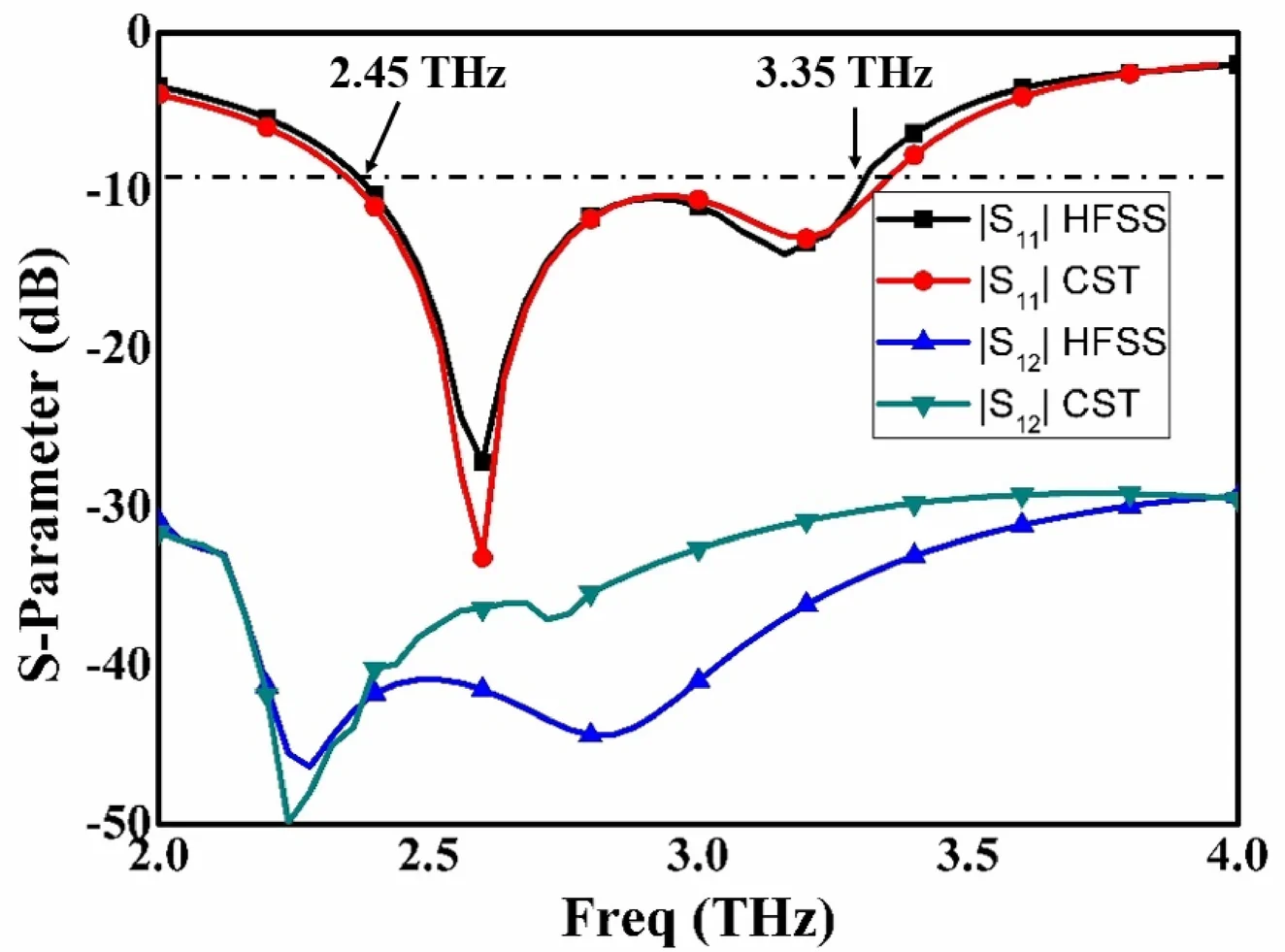
Comparison of S-parameter variations attained from CST/HFSS.

**Fig. 15:**
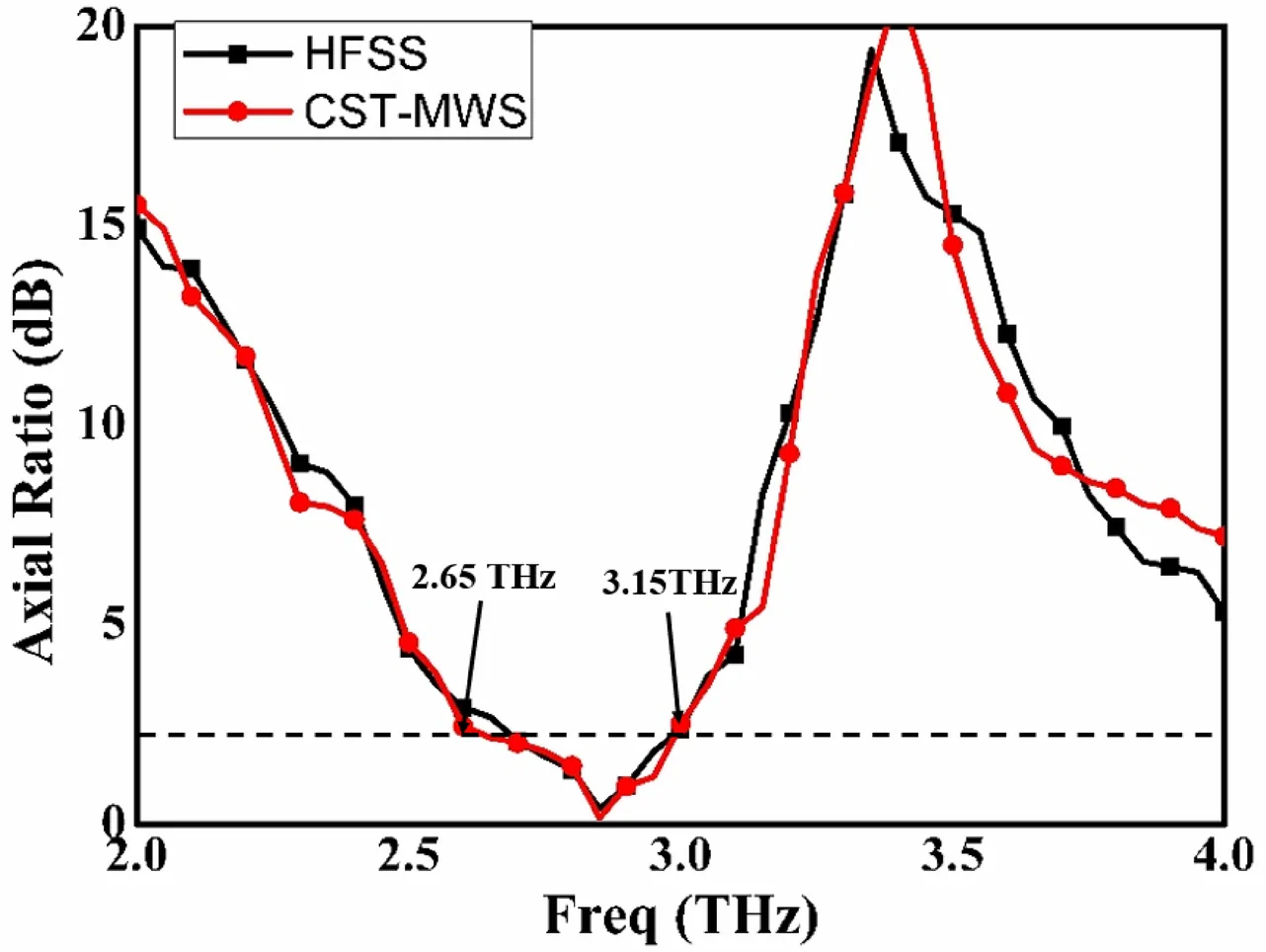
Comparison of AR variations attained from CST/HFSS.

After the thorough design study, the antenna’s optimal performance was confirmed using the CST-MWS platform. The S-parameter findings from the CST and HFSS simulations are shown in Fig. 14. The findings’ dependability is confirmed by the strong agreement between |S_11_| and |S_12_| curves produced by the two solvers. The suggested antenna achieves an isolation level of above 30 dB while continuing to operate well in the 2.45–3.35 THz region. The axial ratio fluctuation anticipated by both HFSS and CST is shown in Fig. 15. The CST-based results, which show circular polarization in 2.65–3.15 THz frequency band, support the HFSS findings.

**Fig. 16:**
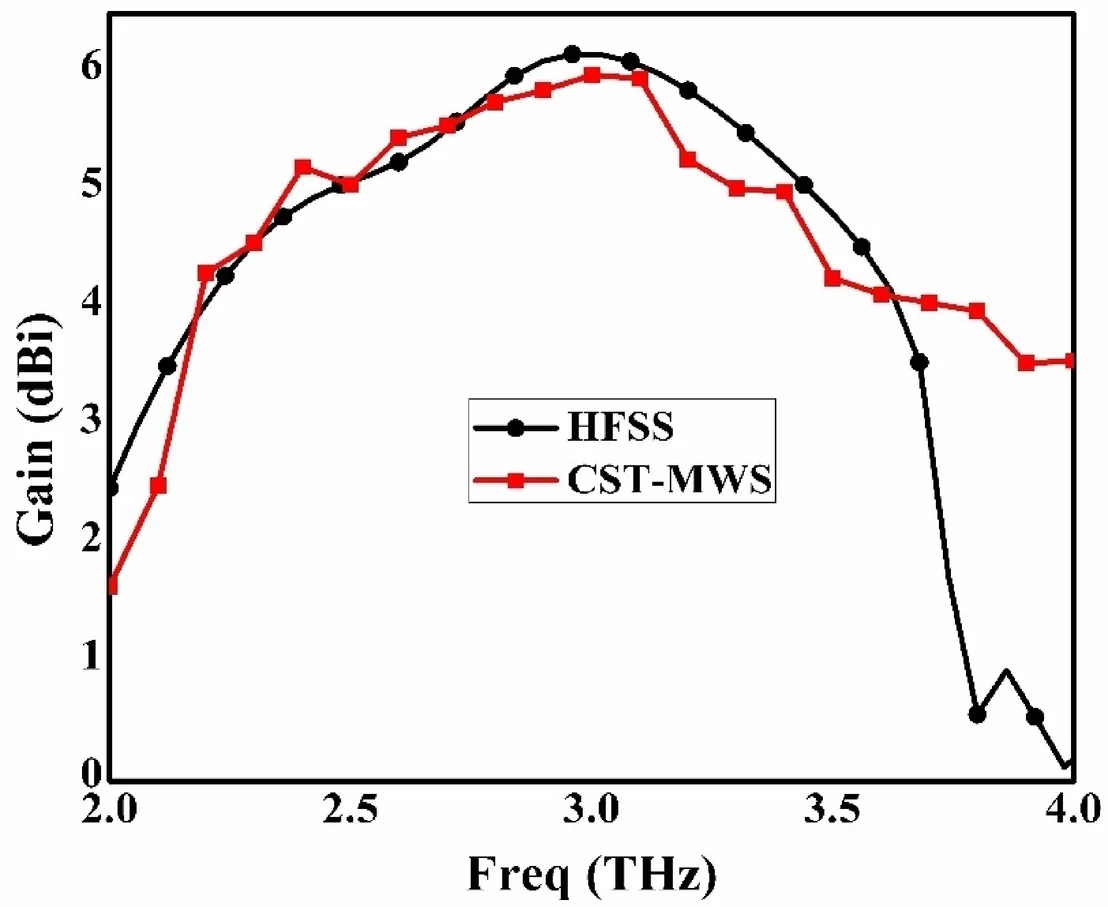
Comparison of Gain variations attained from CST/HFSS.

**Fig. 17:**
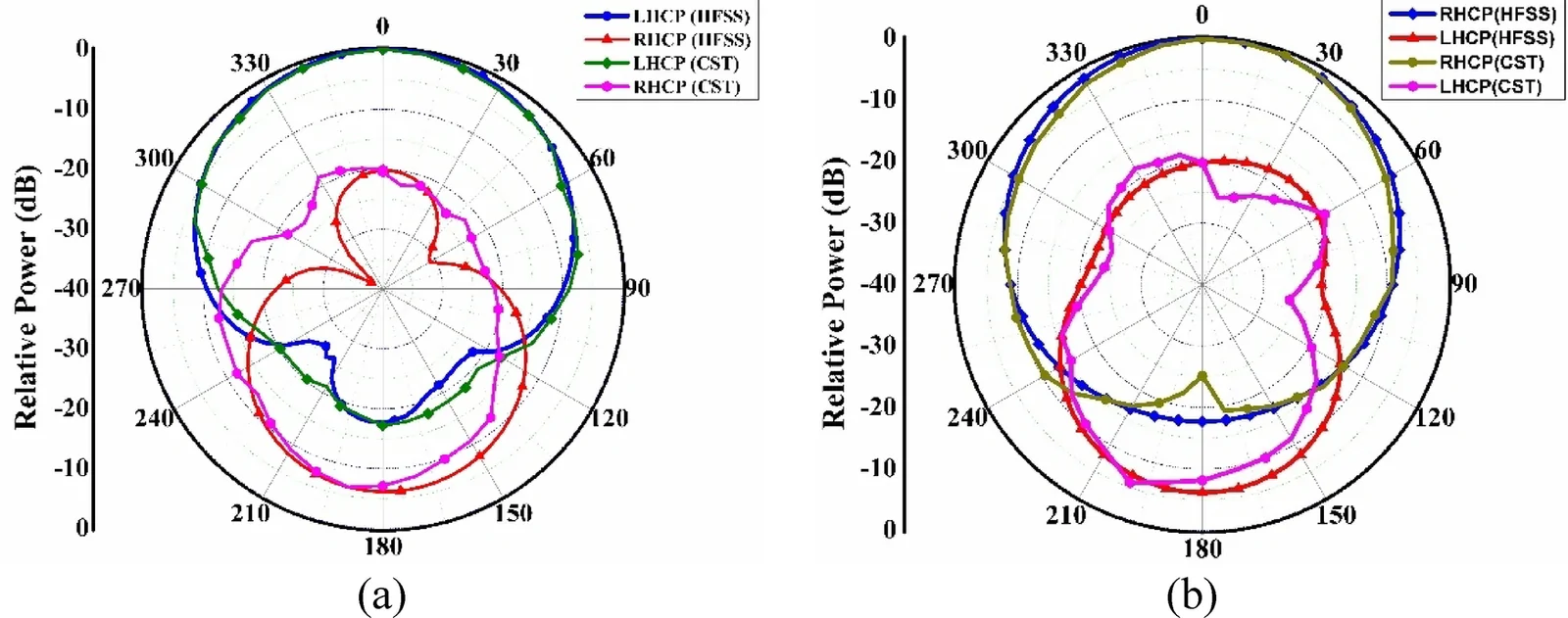
LHCP and RHCP radiation variation in XZ-plane at 2.9 THz: (**a**) Port-1 (**b**) Port-2.

Figure 16 displays the broadside gain variation derived from the CST/HFSS simulations, demonstrating the high degree of agreement between the two solvers. 6.0 dBi is the maximum broadside gain. The LHCP and RHCP radiation patterns for ports 1 and 2 at 2.9 THz in the XZ-plane are shown in Fig. 17. One may make two important observations: (i) LHCP with port-1 and RHCP with port-2, and (ii) both ports reach their maximal radiation in the + z direction. These findings verify that the basic hybrid mode is supported by the suggested antenna. The compact geometry and metasurface-assisted structure may introduce secondary back radiation components (dominant LHCP component observed in the back-lobe region) at THz frequencies due to diffraction and surface-wave scattering. However, the primary forward radiation maintains the desired polarization purity.

**Fig. 18:**
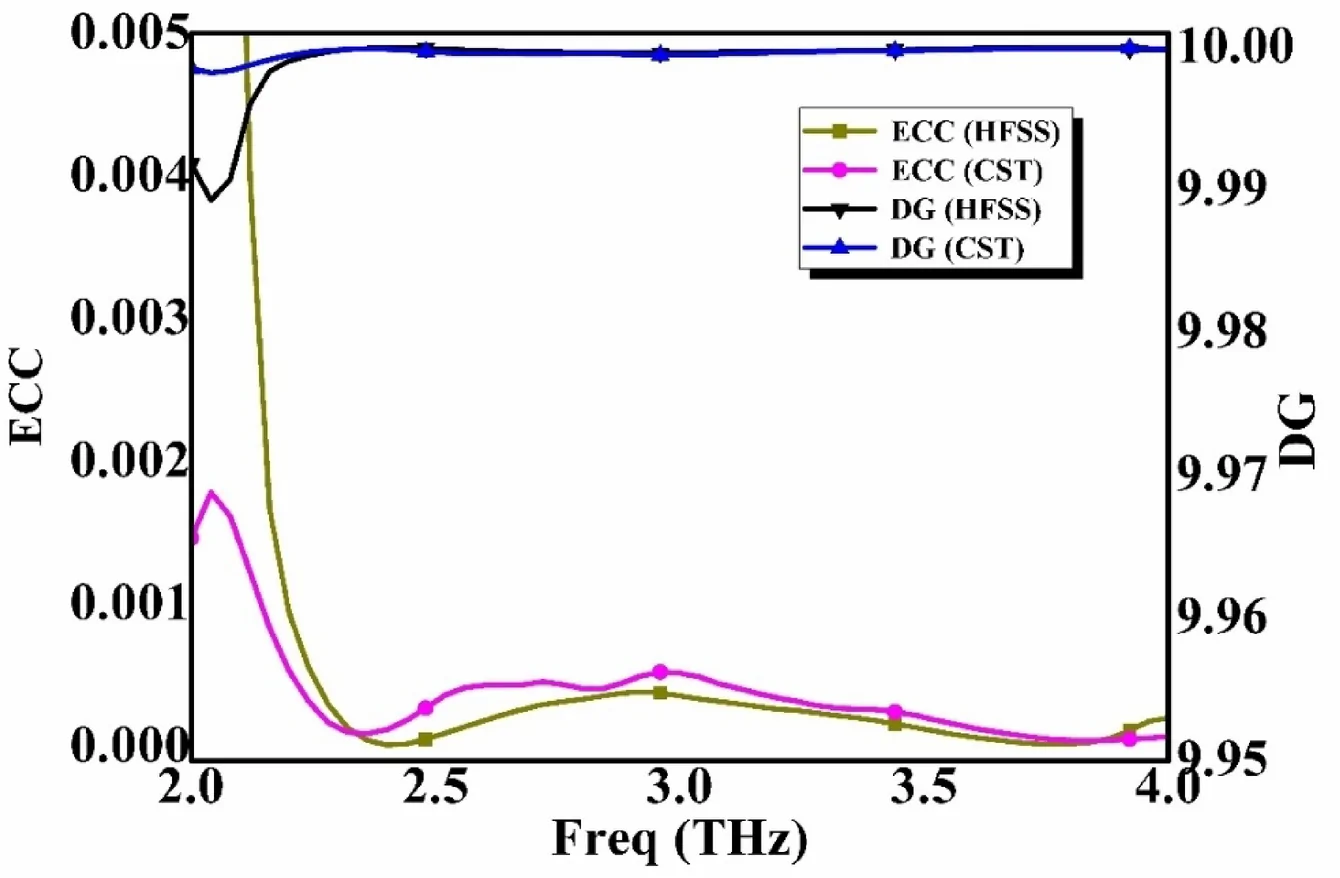
Comparison of ECC/DG variations attained from CST/HFSS.

The planned antenna’s bandwidth and isolation are compared to those of previously published 2-port THz antennas in Table 2. The findings show that, in comparison to current THz MIMO designs, the developed 2-port THz antenna provides greater isolation, better impedance, and AR bandwidth. A major factor in this performance improvement is the reduction of In and Out Band RCS reduction. The Envelope Correlation Coefficient (ECC) and Diversity Gain (DG), two diversity features of planned 2-port antenna, are shown in Fig. 18. Effective isolation is often indicated by ECC values below 0.2, which measure how similar the ports 1 and 2 are. DG, which has an ideal value of 10 dB, shows how well a multi-port antenna performs in fading situations^31^. The suggested design confirms its applicability for diversity applications by achieving an ECC below 0.1 and a DG of around 10 dB over the operating range, as seen in Fig. 18.

**Table 2:** Performance comparison of the planned THz MIMO antenna with existing designs in terms of BW, coupling, and RCS reduction.

References	Electrical size ($\lambda_{0} \times \lambda_{0}$)	Impedance FBW (%)	3-dB AR FBW (%)	Isolation (dB)	Polarization type	Tunability	Low-RCS	Gain (dBi)
^9^	0.82 × 0.82	13.4	8.9	20	CP	Yes	No	5.2
^10^	0.75 × 0.75	21.5	10.7	25	LHCP	Yes	No	6.1
^11^	0.69 × 0.69	22.2	4.8	20	Pattern Diversity	Yes	No	5.8
^12^	0.71 × 0.71	15.0	7.7	20	RHCP	Yes	No	5.9
^13^	0.73 × 0.73	20.0	4.3	25	CP	Yes	No	6.0
^23^	0.78 × 0.78	24.8	9.2	24	CP	Yes	No	6.5
^24^	0.66 × 0.66	28.1	10.1	26	LHCP	Yes	No	6.8
^25^	0.64 × 0.64	30.2	11.3	27	Dual-CP	Yes	No	6.9
Proposed Work	0.73 × 0.73	32.5	10.8	> 30	LHCP/RHCP	Yes	Yes	7.1

**Fig. 19:**
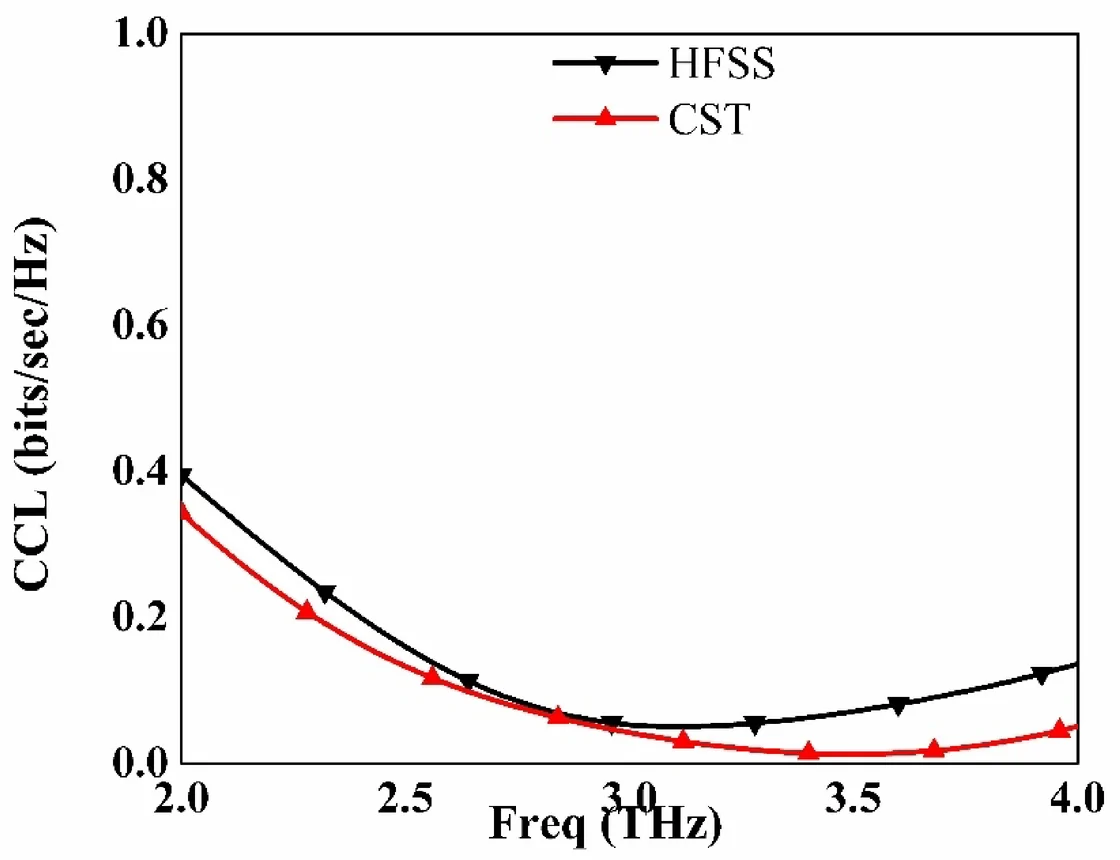
Comparison of CCL variations attained from CST/HFSS.

**Fig. 20:**
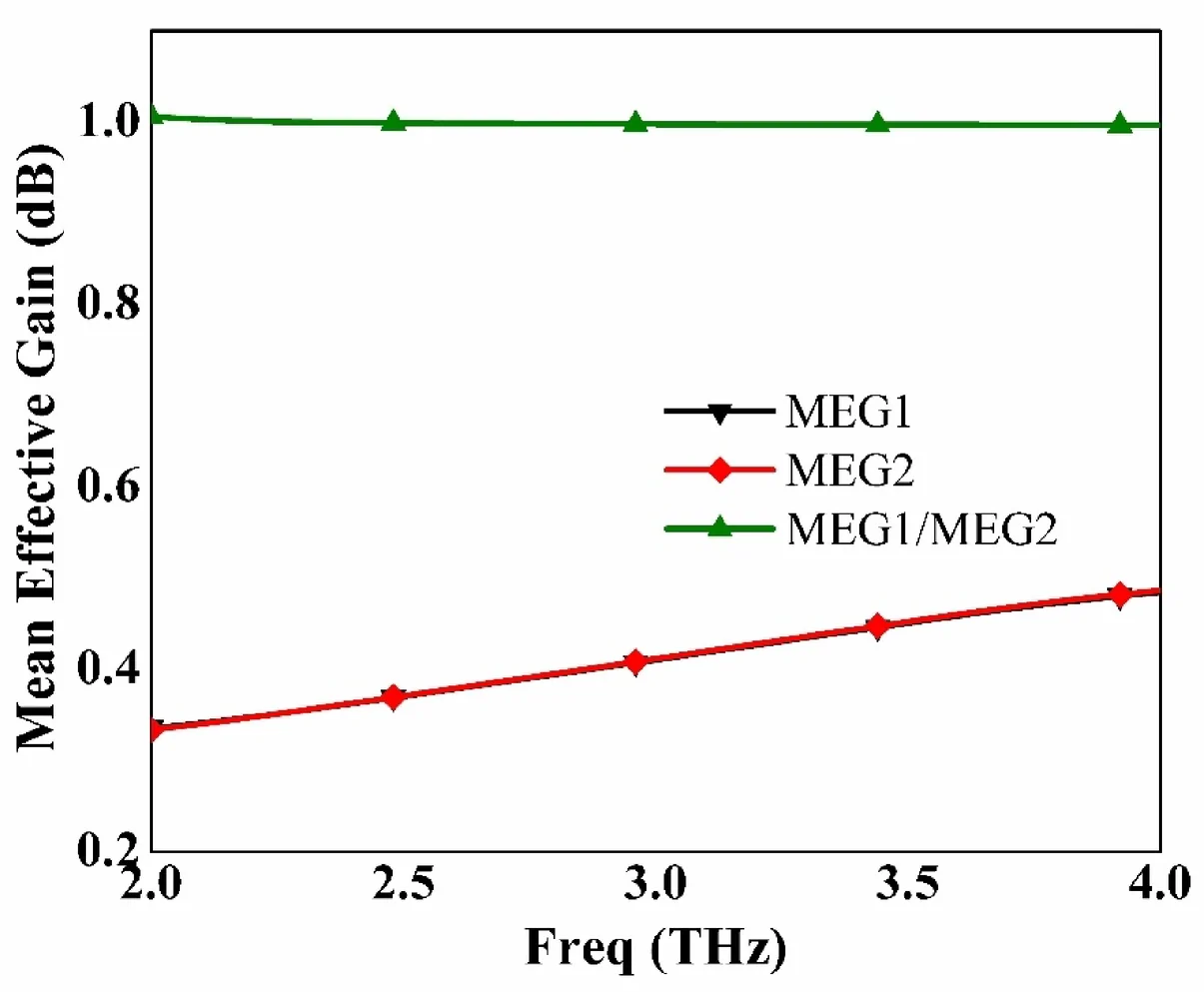
Comparison of MEG variations attained from CST/HFSS.

The extra diversity metrics, mean effective gain (MEG) and channel capacity loss (CCL), are shown in Figs. 19 and 20, respectively. In a MIMO system, CCL stands for the channel capacity loss that happens during signal transmission. The CCL value should stay below 0.4 bits/s/Hz for an effective MIMO antenna [30]. The suggested antenna keeps the CCL considerably below this threshold across the working frequency range, as shown in Fig. 19, demonstrating its appropriateness for MIMO communication applications.

The average received power of a multi-port antenna in comparison to a single-port radiator is shown by the MEG parameter^31^. Figure 20 shows that the suggested antenna’s MEG value stays below 3 dB across the operating band, confirming adequate diversity performance and balanced power.

## Practical fabrication and characterization considerations

The proposed THz antenna can be fabricated using standard microfabrication and semiconductor processing techniques. Initially, the SiO_2_ substrate can be prepared using conventional wafer processing methods. The metallic feed line, ground plane, and SRR-based metasurface may be realized through thin-film metal deposition techniques such as electron-beam evaporation or sputtering followed by photolithographic patterning.

The cylindrical silicon dielectric resonator can be fabricated using micromachining or precision etching techniques and positioned accurately over the aperture-coupled feeding structure. The graphene layer may be synthesized using chemical vapor deposition (CVD) and transferred onto the dielectric resonator surface using standard graphene transfer techniques.

For practical tunability implementation, a thin dielectric spacer layer such as Al_2_O_3_ or HfO_2_ may be deposited beneath the graphene layer to realize electrostatic gating through bias electrodes. The bias network can be integrated using high-resistance bias lines and RF choke structures to minimize interference with THz signals.

The antenna performance can be experimentally characterized using a terahertz vector network analyser (THz-VNA), near-field THz scanning system, or THz time-domain spectroscopy (THz-TDS) setup. Reflection coefficient, isolation, gain, axial ratio, and radiation characteristics can be measured using standard THz measurement procedures in an anechoic chamber environment.

The approximate research-level fabrication cost of the proposed prototype may range from 300 to 800 USD depending on substrate processing, graphene synthesis, cleanroom facilities, and THz characterization availability. The major cost contributors include graphene deposition, precision lithography, and THz measurement instrumentation.

## Conclusion

This study presents a 2-port Si–graphene-built antenna operating in the THz range. By exciting dual orthogonal hybrid modes, the antenna achieves an operational bandwidth spanning 2.45–3.35 THz. A graphene coating over the cylindrical silicon dielectric resonator enables frequency reconfigurability through chemical potential tuning. The proposed graphene tuning mechanism is based on electrostatic gating, which can be practically realized using thin high-k dielectric layers and localized bias electrodes to avoid excessive bias voltage requirements associated with thick substrate configurations. The stair-slot design generates circularly polarized (CP) waves within 2.65–3.15 THz range, while the implementation of polarization diversity significantly improves isolation, exceeding 30 dB across the band. Furthermore, SRR are employed to reduce the in and out band RCS. These combined attributes make the proposed radiator a strong candidate for THz wireless communication applications used in stealth technology.

## Data Availability

The datasets used and/or analyzed during the current study are available from the corresponding author on reasonable request.
